# Hsp90 up-regulates PD-L1 to promote HPV-positive cervical cancer via HER2/PI3K/AKT pathway

**DOI:** 10.1186/s10020-021-00384-2

**Published:** 2021-10-19

**Authors:** Jie Zeng, Si-Li He, Li-Jie Li, Chen Wang

**Affiliations:** 1 Pharmacy Intravenous Admixture Services, The Third Xiangya Hospital of Central South University, Changsha, 410013 Hunan Province People’s Republic of China; 2 Department of Gynecology and Obstetrics, The Third Xiangya Hospital of Central South University, No.138, Tongzipo Road, Changsha, 410013 Hunan Province People’s Republic of China

**Keywords:** HPV16, Cervical cancer, Hsp90, HER2, PD-L1

## Abstract

**Background:**

HPV16 is the predominant cancer-causing strain that is responsible for over 50% of all cervical cancers. In this study, we aim to investigate the therapeutic effect of heat shock protein 90 (Hsp90) knockdown on HPV16^+^ cervical cancer progression and the underlying mechanism.

**Methods:**

The transcript and protein expression of Hsp90 in normal cervical and HPV16^+^ cervical cancer tissues and cell lines were detected by qRT-PCR, immunohistochemistry staining and Western blot. Hsp90 knockdown clones were established using HPV16^+^ cervical cancer cell line Caski and SiHa cells. The effect of Hsp90 knockdown on HER2/PI3K/AKT pathway and PD-L1 expression was characterized using qRT-PCR and Western blot analysis. Cell proliferation and migration were determined using MTT and transwell assays. Using mouse xenograft tumor model, the impact of Hsp90 knockdown and PD-L1 overexpression on tumor progression was evaluated.

**Results:**

Hsp90 expression was up-regulated in HPV16^+^ cervical cancer tissues and cells. Knockdown of Hsp90 inhibited proliferation and migration of Caski and SiHa cells. PD-L1 expression in cervical cancer tissues was positively correlated with Hsp90 expression, and Hsp90 regulated PD-L1 expression via HER2/PI3K/AKT signaling pathway. The results of mouse xenograft tumor model demonstrated Hsp90 knockdown suppressed tumor formation and overexpression of PD-L1 simultaneously eliminated the cancer-suppressive effect of Hsp90 knockdown.

**Conclusion:**

In this study, we demonstrated a promising tumor-suppressive effect of Hsp90 knockdown in HPV16^+^ cervical cancers, and investigated the underlying molecular pathway. Our results suggested that Hsp90 knockdown holds great therapeutic potential in treating HPV16^+^ cervical cancers.

## Background

Human papillomavirus (HPV) infection is causing over 90% of cervical cancers worldwide. There are over 100 different strains of HPV. Although most of the strains are low risk, several strains can be the prevalent causes of cervical cancers, including HPV16, 18, 31, 33 and 45 (Clifford et al. 2003; Liou et al. 2015). Persistent HPV16 infection causes over 50% of all cervical cancers, making it the most predominant high risk strain (Mirabello 2017). HPV16 infection may progress undetectably within 1–2 years, which makes it challenging for early diagnosis of cervical cancer. Therefore, exploring potential treatment for HPV16^+^ cervical cancer is in demand.

Heat shock protein 90 (Hsp90) is an essential chaperone protein that assists proper protein folding to regulate various biological processes including cell proliferation, apoptosis, migration and signaling (Cox and Johnson 2018; Zeng et al. 2015; Am et al. 2012). Notably, studies have revealed cancer cells utilize Hsp90 to chaperone the folding of oncogenic mutant proteins to stabilize their functions, inspiring the exploration of cancer therapies targeting Hsp90 (Li et al. 2011a). Schwock et al. discovered Hsp90 inhibition induced apoptosis of cervical carcinoma cells in vitro and in vivo (Schwock et al. 2008). Ajiro and Zheng investigated the potential molecular mechanism of Hsp90 in HPV16^+^ cervical cancer, and found Hsp90 and Grp78 chaperoned key proteins E6 and E7 in HPV16^+^ Caski cells (Ajiro and Zheng 2015). However, the role of Hsp90 in regulating other critical signaling pathways in cervical cancers is unclear.

Human epithermal growth factor receptor 2 (HER2/ErbB2) is the protein that promotes the growth of various cancer cells and leads to poor prognosis (Jordan et al. 2016; Wen et al. 2000). Overexpression of HER2 has been found in several solid tumors, especially breast cancer (Loibl and Gianni 2017; Yuan et al. 2020). In cervical carcinomas the ratio of HER2 overexpressing tumors various from 8 to 77% evaluated by diverse methods, and high HER2 expression is reported to be associated with poor prognosis (Chavez-Blanco et al. 2004). It is known that Hsp90 chaperons and stabilizes HER2 in some tumors (Patel et al. 2013), however, whether Hsp90 can function through HER2 in modulating cervical cancer progress remains unknown.

Programmed death-ligand 1 (PD-L1) is a transmembrane protein, which is responsible for the immune suppressive microenvironment in tumors (Giatromanolaki et al. 2019; Zou et al. 2016; Huang et al. 2020). The activation of PI3K/AKT signaling pathway in cancers promotes PD-L1 expression (Atefi et al. 2014; Lastwika et al. 2016), and HER2 overexpression activates PI3K/AKT signaling pathway (Vernieri et al. 2020; Li et al. 2011b). This interaction leads to a positive feedback of PD-L1 signaling, which further enhances the immune suppressive microenvironment in tumors.

In this study, we targeted Hsp90 in HPV16^+^ cervical cancer, and investigated its effect on HER2/PI3K/AKT signaling and PD-L1 expression. We hypothesize Hsp90 stabilizes HER2 to activate PI3K/AKT signaling, promote PD-L1 expression, and enhance cervical cancer cell proliferation and migration in vitro and tumor progress in vivo. To our knowledge, it is the first to report this particular signaling pathway in cervical cancer, providing potential therapeutic targets for HPV16^+^ cervical cancer treatment.

## Materials and methods

### Clinical sample collection

A total number of 87 normal and cervical cancer tissues were obtained from the Third Xiangya Hospital of Central South University. The specimens included 36 normal cervical tissues and 52 cervical cancer tissues. All samples were screened for HPV infection, and 25 HPV^−^ normal cervical tissues were chosen as control. HPV^+^ samples in cervical cancer tissues were processed for further verification of HPV strains. HPV16^+^ samples were verified using the primers specifically HPV-16 E6 and E7 transcripts (Gao et al. 2013). A total number of 38 HPV16^+^ samples were chosen for this study. The clinicopathologic parameters of the samples were shown in Table 1. All procedures were approved by the Ethical board at the Third Xiangya Hospital of Central South University.

**Table 1:** Relationship between HSP90 and clinicopathological parameters in HPV16^+^ cervical cancer patients

Characteristics	Numbers	HSP90		p value
		Low	High	
Total cases	38	19	19	
Age				
≤ 55	18	10	8	0.7459
> 55	20	9	11	
Tumor size (cm)				
≤ 4.0	10	8	2	0.0232
> 4.0	28	9	19	
Histology				
Squamous	21	9	12	0.5148
Adenocarcinoma	17	10	7	
FIGO stage				
Ib–IIa	12	8	4	0.0326
IIb–IIIa	26	7	19	
Lymph node metastasis				
Yes	27	7	20	0.0117
No	11	8	3	

### Cell culture

Human HPV16+cervical cancer cell lines Caski and SiHa, and normal cervical epithelial cell line H8 cells were purchased from American Type Culture Collection (ATCC, Manassas, VA, USA). Cells were cultured in Dulbecco’s Modified Eagle Medium (DMEM) (Gibco) supplemented with 10% heat-inactivated fetal bovine serum (FBS) and 1 × Penicillin–Streptomycin (Thermo Fisher Scientific) in 5% CO_2_ atmosphere at 37 °C.

### CRISPR–Cas9-mediated gene disruption

The sgRNA targeting PD-L1 (5′- GGTTCCCAAGGACCTATATG- 3′) was cloned into pSpCas9(BB)-2A-GFP (pX458). Caski and SiHa cells were transiently transfected with pX458-PD-L1 or the pX458 empty vector using Lipofectamine 3000 (Life Technologies). PD-L1 null clones were obtained by GFP+ single cell cloning.

### Quantitative real time polymerase chain reaction (qRT-PCR)

The total RNA was isolated from cervical tissues and cells using TRIzol reagent (Invitrogen). The mRNA was quantified by nanodrop 2000 (Thermo Fisher Scientific) and reverse transcribed using High Capacity cDNA kit (Thermo Fisher Scientific). The relative expression was determined using SYBR green Master mix (Thermo Fisher Scientific) by real-time polymerase chain reaction (RT-PCR) calculated using 2^−ΔΔCt^ method. The primer sequences of all genes detected and the reference gene were listed in Table 2.

**Table 2:** The primer sequences

Name	Forward (5′–3′)	Reverse (5′–3′)
HER2	AAAGGCCCAAGACTCTCTCC	CTCTGGGTTCTCTGCCGTAG
PD-L1	ACCACCACCAATTCCAAGAG	GATGGCTCCCAGAATTACCA
Hsp90	TGGACAGCAAACATGGAGAG	AGACAGGAGCGCAGTTTCAT
GAPDH	CCAGGTGGTCTCCTCTGA	GCTGTAGCCAAATCGTTGT

### Western blot

Tissues and cells were lysed using RIPA lysis buffer (Sigma Aldrich) and the total proteins were quantified using PierceTM BCA protein assay kit (Thermo Fisher Scientific). Proteins were separated by sodium dodecyl sulphate–polyacrylamide gel electrophoresis (SDS-PAGE) and subsequently transferred onto polyvinylidene difluoride membranes. The membranes were blocked using 5% bovine serum albumin (BSA) for 1 h, and incubated overnight at 4 °C with the primary antibodies, including anti-Hsp90 (Abcam ab13492, 1:500), anti-PD-L1 (Abcam ab205921, 1:500), anti-HER2 (Abcam ab237715, 1:500), anti-p-HER2 (Abcam ab108371, 1:500), anti-AKT (Abcam ab131168, 1:1000), anti-p-AKT (Abcam ab38449 1:500), and tubulin as internal control (Abcam ab6160, 1:1000). Next, the membranes were washed using TBST for five times and then incubated at room temperature with HRP-conjugated secondary antibodies for 1 h. Finally, the membranes were visualized and the protein intensities were detected using ECL chemiluminescence reagents (Pierce).

### Cell transfection and treatment

Small interferon RNA against Hsp90, HER2 and its negative control were obtained from Ambio (Austin, TX). The cDNA vector encoding human PD-L1 and the negative control were from OriGene (Rockville, MD). Transfection of Caski and SiHa cells were accomplished using Lipofectamine 3000 (Invitrogen, Carlsbad, CA) according to the manufacturer’s protocols. Stable transfected Caski and SiHa cells were generated using negative control against Hsp90 (sh-NC), sh-Hsp90#1, sh-Hsp90#2, sh-Hsp90#1+vector, sh-Hsp90#1+PD-L1, sh-HER2#1 or sh-HER2#2. After 48 h transfection, cells were treated with AKT agonist SC79 (1 µg/mL) for 1 h.

### MTT assay

Caski and SiHa cells after stable transfection were replated in 96-well plates (2 × 10^4^ cells/well) and cultured for 0, 24, 48, 72 and 96 h. A total volume of 20 μL MTT solution was added to each well, and the cells were further incubated at 37 °C for 4 h. After adding 150 μL DMSO to each well, the absorbance was measured at OD490 nm by spectrophotometer.

### Transwell assay

Cell migration was detected using transwell assay. The cells from each group were suspended in serum free medium at the density of 5 × 10^5^ cells/mL, and were added into the top chamber of the transwell with 8.0 μm pore size polycarbonate membrane (Corning, USA). Complete medium with 10% fetal bovine serum (FBS) was added into the lower chamber. The cells were allowed to migrate for 48 h, and the migrated cells on the chamber were fixed and stained with crystal violet and imaged using the microscope (Zeiss, Germany). Migrated cell number were counted from at least five photos randomly taken from each well.

### Immunohistochemistry

Tumor tissues were fixed overnight in 10% formalin (Sigma Aldrich), and dehydrated using ethanol with gradient concentrations. The tumors were embedded in parrafin and cut into 5 μm thick sections. The sections were dried and deparaffinized before antigen retrieval using Citrate buffer at pH 6. The sections were then incubated with primary antibodies against Hsp90 (Abcam ab203085), and Ki-67 (Abcam ab15580) overnight at 4 °C. After staining with HRP as secondary antibody (Abcam ab6721), the sections were counter stained with Hematoxylin and mounted with Permount Mounting Medium (Thermo Fisher Scientific).

### Animal xenograft tumor model

The animal experiments were performed in accordance with the guidelines of the Animal Care and Use Committee at the Third Xiangya Hospital of Central South University. A total number of 30 6-week-old female nude mice (BALB/c) were purchased from SJA Laboratory Animal Co., Ltd. (Hunan, China), and housed in the facility for at least 5 days before the experiment. The mice were equally divided five group for injecting the indicated SiHa cells (1 × 10^6^): control; sh-NC; sh-Hsp90; sh-Hsp90+ vector; sh-Hsp90+ PD-L1. The cells were suspended in 100 µL PBS and then injected subcutaneously into the mice. For tail vein injection, the indicated cells in 100 μL of medium were gently injected into mice tail vein while mice were placed in the prone position. The mice were closely monitored daily and the tumor volume was measured using digital calipers at day 0, 7, 14, 17, 21, 24, and 28. The tumor volume (V) was calculated using the formula V = (S × S × L) × 0.5, where S and L were the short and long dimensions of the tumor, respectively. At day 28, the mice were euthanized, and the tumors were excised and weighed. The harvested tumors were cut into pieces for different characterization.

### Hematoxylin and eosin (H&E) staining

The tissue sections were dewaxed and rehydrated, following by staining with Mayer’s hematoxylin solution for 30 s and 1% eosin Y solution for another 30 s. Then the slides were dehydrated and mounted for photo taking. Hematoxylin and eosin solution were products of Sigma-Aldrich (St. Louis MO, USA).

### Statistical analysis

All experiments were performed at least three times independently with n = 3 in each group. All data were presented as mean ± standard deviation. Statistical analysis was performed using Student’s *t* test between two groups, and one-way analysis of variance (ANOVA) among three or more groups. *P* < 0.05 was considered statistically significant.

## Results

### Hsp90 expression was upregulated in HPV-positive cervical cancer tissues and cell lines

**Fig. 1:**
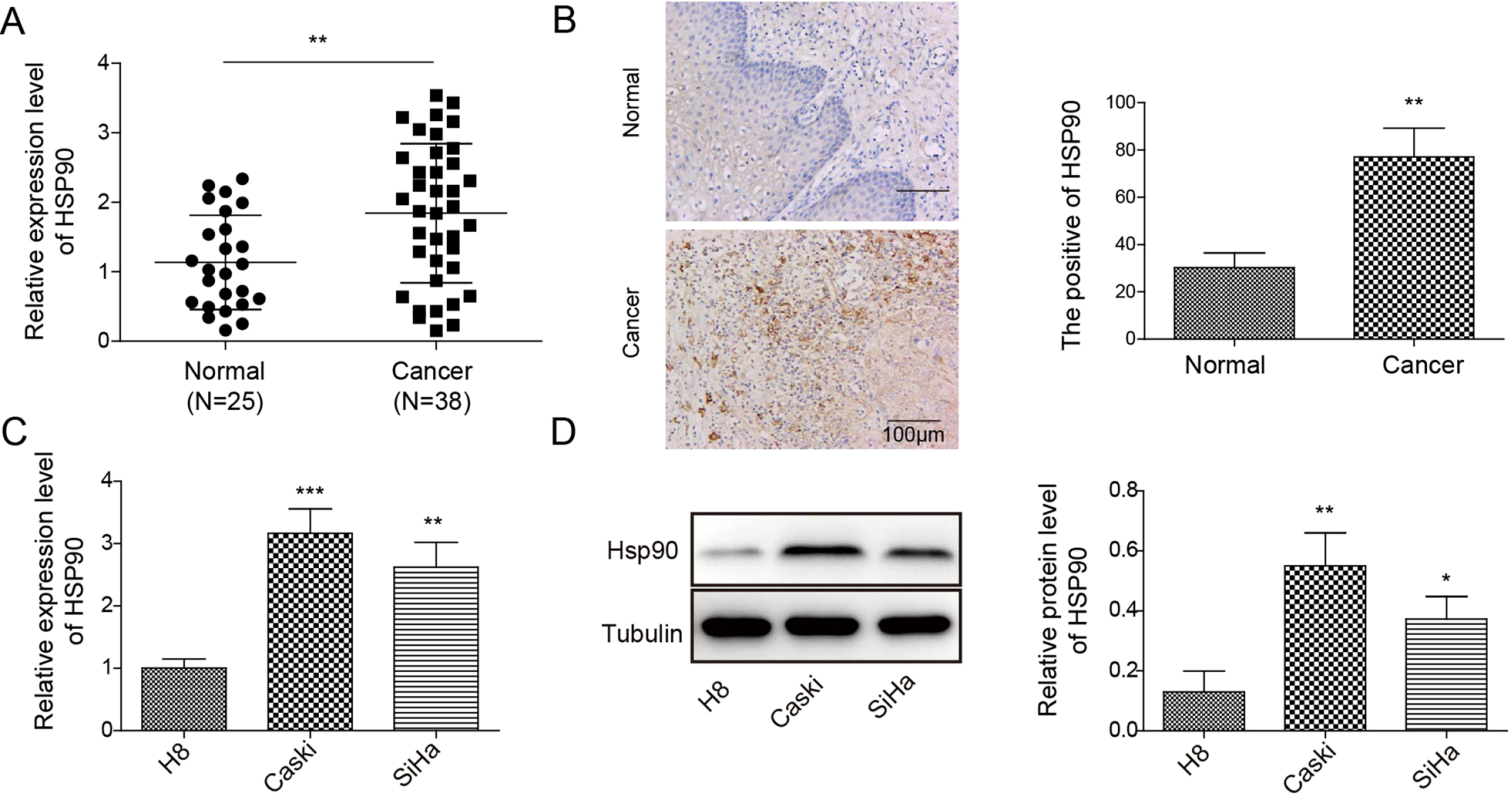
The Hsp90 expression was increased in HPV16^+^ cervical cancer. **A** *Hsp90* gene expression in normal and cancer tissues. **B** IHC staining of Hsp90 in normal and caner tissues. **C** *Hsp90* gene expression in normal (H8) and cancer (Caski and SiHa) cervical epithelial cell lines. **D** Western blot of Hsp90 expression in normal (H8) and cancer (Caski and SiHa) cervical epithelial cell lines. Clinical samples: N = 25 in normal tissue group, N = 38 of cancer tissue group. **p < 0.01. Cell culture study: N = 3 in each group. *p < 0.05, **p < 0.01 or ***p < 0.001

We collected 25 HPV16^−^ normal tissues and 38 HPV16^+^ cancer tissues, and evaluated *Hsp90* gene expression. The qRT-PCR result showed HPV16^+^ cancer tissues had significantly higher *Hsp90* gene expression as compared to the HPV^−^ normal tissues (Fig. 1A). As shown in Table 1, we found that the patients with high expression of Hsp90 displayed bigger tumor size, higher FIGO grade, and more lymph node metastasis compared with that of low expression of HSP90. IHC staining of Hsp90 also demonstrated a markedly increase of positive staining in HPV16^+^ cancer tissues in comparison to normal tissues (Fig. 1B). The Hsp90 expression was further investigated in normal cervical epithelial cell line H8 and HPV16^+^ cervical cancer epithelial cell lines Caski and SiHa. The results from qRT-PCR and Western blot demonstrated that both cancer cell lines had significantly higher transcript and protein expressions of Hsp90 as compared to H8 cells (Fig. 1C, D). These results suggested that Hsp90 expression was closely associated with HPV16^+^ cervical cancers in both clinical cancer tissues and cancer cell lines.

### Knockdown of Hsp90 inhibited the proliferation and migration of cervical cancer epithelial cells

**Fig. 2:**
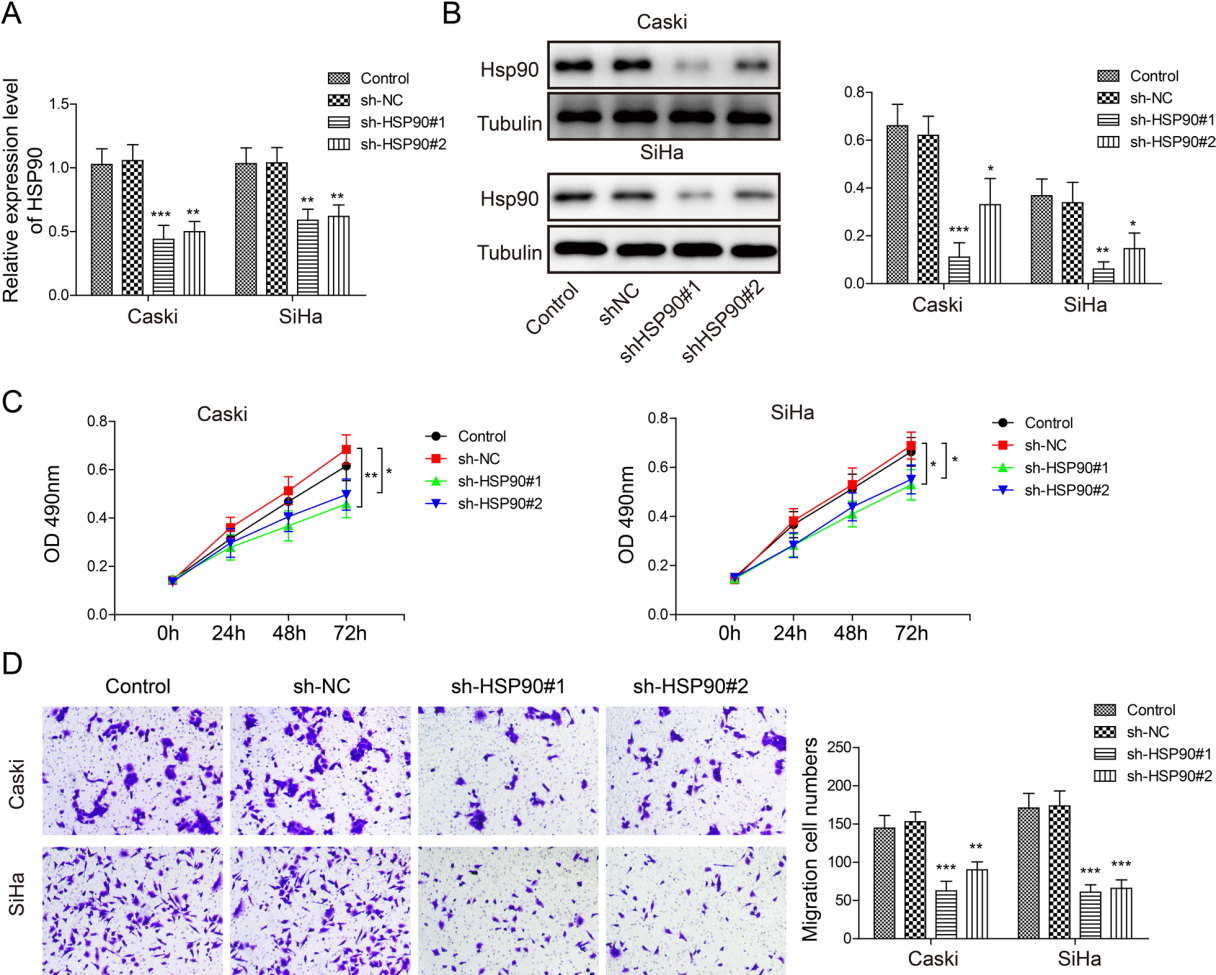
Hsp90 knockdown inhibited HPV16^+^ cervical cancer cell proliferation and migration. In Caski and SiHa cell culture, **A** *Hsp90* gene expression in control, shNC, sh-Hsp90#1 and sh-Hsp90#2 groups. **B** Western blotting image and analysis of Hsp90 expression in control, shNC, sh-Hsp90 groups. **C** MTT assay evaluating cell proliferation in control, shNC, sh-Hsp90#1 and sh-Hsp90#2 groups. **D** transwell assay evaluating cell migration in control, shNC, sh-Hsp90#1 and sh-Hsp90#2 groups. N = 3 in each group. *p < 0.05, **p < 0.01 or ***p < 0.001

We established Hsp90 knockdown clones using HPV16^+^ cervical cancer epithelium cell lines Caski and SiHa. The qRT-PCR and Western blot results showed the *Hsp90* gene expression was significantly suppressed in sh-Hsp90#1 and sh-Hsp90#2 groups in both cell lines as compared to wild type control and sh-NC (Fig. 2A, B), indicating the Hsp90 knockdown was successful. MTT assay results demonstrated that the cell proliferation was inhibited in sh-Hsp90#1 and sh-Hsp90#2 groups as compared to control and sh-NC group (Fig. 2C). The migration ability of Caski and SiHa cells was also markedly inhibited by sh-Hsp-90#1 and sh-Hsp90#2, as the migrated cell numbers through the transwell were significantly lower than the controls and sh-NC groups (Fig. 2D). The results above indicated that Hsp90 knockdown suppressed the proliferation and migration of HPV16^+^ cervical cancer cells.

### *Hsp90 positively regulated PD-L1 and HER2 expression in HPV16*^+^*cervical cancer tissues and cell lines*

**Fig. 3:**
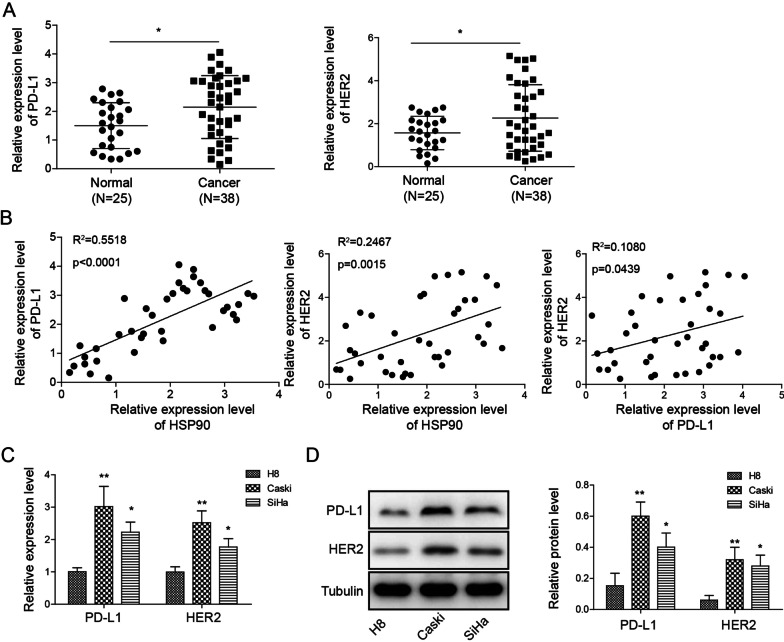
Hsp90 correlated with PD-L1 and HER2 expression in HPV16^+^ cervical cancer. **A** *PD-L1* and *HER2* gene expression in normal and cancer tissues. **B** correlation analysis among Hsp90, PD-L1 and HER2 expression in normal and cancer tissues. **C** *PD-L1* and *HER2* gene expression in normal (H8) and cancer (Caski and SiHa) cervical epithelial cell lines. **D** Western blot of PD-L1 and HER2 expression in normal (H8) and cancer (Caski and SiHa) cervical epithelial cell lines. Clinical samples: N = 25 in normal tissue group, N = 38 of cancer tissue group. *p < 0.05. Cell culture study: N = 3 in each group. *p < 0.05 or **p < 0.01

To investigate the association among Hsp90, PD-L1 and HER2 in HPV16^+^ cervical cancer, we evaluated the gene expression of *PD-L1* and *HER2* in the 25 HPV^−^ normal tissues and 38 HPV16^+^ cervical cancer tissues. The results shown that *PD-L1* and *HER2* gene expression were elevated in HPV16^+^ cervical cancer tissues (Fig. 3A). The *Hsp90*, *PD-L1* and *HER2* gene expression data were analyzed using Pearson correlation analysis, and the result showed a positive correlation not only between Hsp90 and PD-L1 or HER2, but also between PD-L1 and HER2 (Fig. 3B). In HPV16^+^ cervical cancer line Caski and SiHa, the PD-L1 and HER2 transcript and protein expressions were significantly upregulated as compared to normal epithelial cells H8 (Fig. 3C, D), indicating PD-L1 and HER2 are closely associated with HPV16^+^ cervical cancer.

### *HER2 increased PD-L1 expression *via* PI3K/AKT to promote the proliferation and migration of cervical cancer cells*

**Fig. 4:**
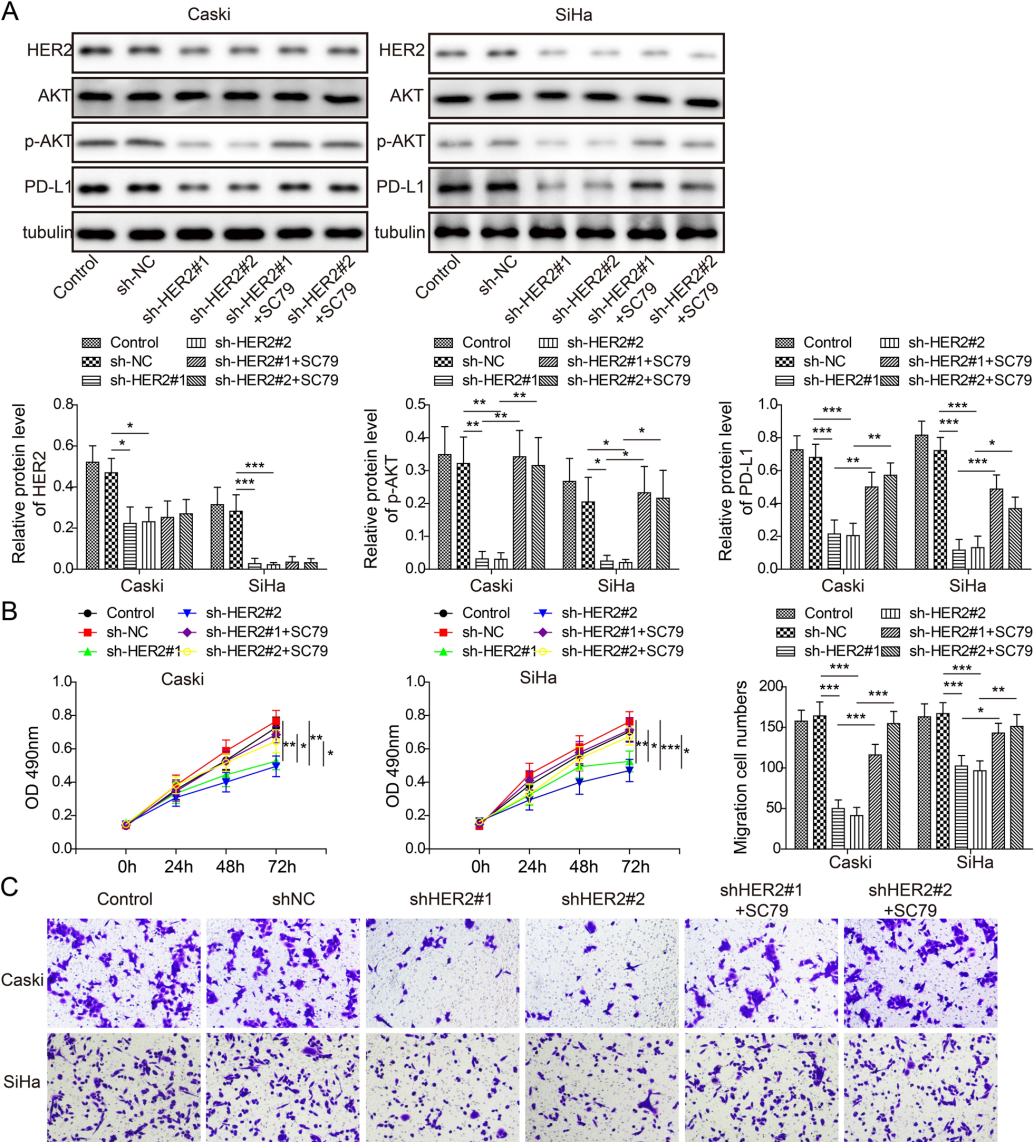
HER2 increased PD-L1 expression via PI3K/AKT to promote the proliferation and migration of cervical cancer cells. **A** Western blotting image and analysis of HER2, AKT, p-AKT. PD-L1 and tubulin expressions in control, sh-NC, sh-HER2#1, sh-HER2#2, sh-HER2#1+SC79, and sh-HER2#2+SC79 treated Caski and SiHa cells. **B** MTT assay evaluating cell proliferation. **C** Transwell assay evaluating cell migration. N = 3 in each group. *p < 0.05, **p < 0.01 or ***p < 0.001

To explore the potential signaling pathway underlying the HER2/PD-L1 correlation, we tested protein expressions in HER2 knockdown cells, and used AKT agonist SC79 as positive control. In both Caski and SiHa cells, downregulated HER2 significantly suppressed the expressions of PD-L1 and decreased ratio of p-AKT/AKT compared to the control and sh-NC, whereas AKT agonist SC79 significantly restored p-AKT level and PD-L1 expressions (Fig. 4A). Consistently, cell proliferation and migration were decreased in HER2 knockdown cervical cancer cells (Fig. 4B, C). SC79 can restore the reduction of cell proliferation and migration caused by HER2 knockdown (Fig. 4B, C). Collectively, these data suggested that HER2 increased PD-L1 expression via PI3K/AKT to promote the proliferation and migration of cervical cancer epithelial cells.

### *Hsp90 promoted the proliferation and migration of cervical cancer cells partly *via* PD-L1*

**Fig. 5:**
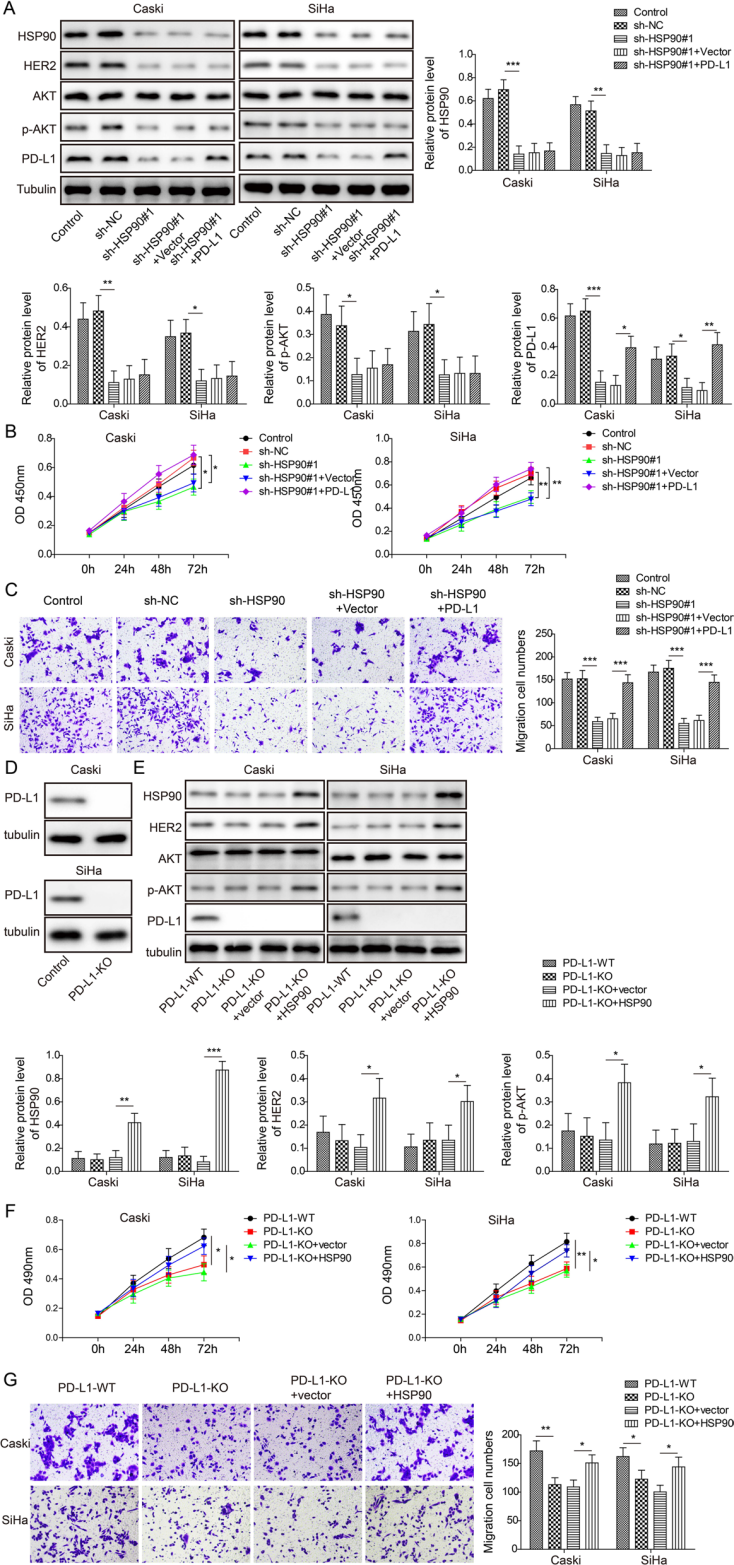
Hsp90 regulated HPV16^+^ cervical cancer cell proliferation and migration via PD-L1. In Caski and SiHa cells **A** Western blotting image and analysis of Hsp90, HER2, AKT, p-AKT, PD-L1 expression in control, sh-NC, sh-Hsp90#1, sh-Hsp90#1+vector, and sh-Hsp90+PD-L1 groups. **B** MTT assay evaluating cell proliferation. **C** Transwell assay evaluating cell migration. **D** Western blotting assay was used to detect the expression of PD-L1 in PD-L1-deficient Caski and SiHa cells. **E** Western blotting image and analysis of Hsp90, HER2, AKT, p-AKT, PD-L1 in PD-L1-deficient or PD-L1-deficient+Hsp90 Caski and SiHa cells. **F** MTT assay evaluating cell proliferation. **G** Transwell assay evaluating cell migration. N = 3 in each group. *p < 0.05, **p < 0.01 or ***p < 0.001

We introduced Hsp-90 knockdown with PD-L1 overexpression clones (sh-Hsp90#1+PD-L1) to investigate the functioning axis between Hsp90 and PD-L1 in HPV16^+^ cervical cancer. Western blotting results showed that PD-L1, HER2 and p-AKT expressions in both Caski and SiHa cells were significantly suppressed by sh-Hsp90 (and sh-HSP90#1+vector), and the expression of PD-L1 was partially restored by PD-L1 overexpression (Fig. 5A). The Caski and SiHa cell proliferation and migration were also significantly suppressed by sh-Hsp90 (and sh-Hsp90+vector) and almost fully restored by sh-Hsp90+PD-L1 (Fig. 5B, C). These results suggested PD-L1 functioned in the downstream of Hsp90, and Hsp90 regulated cell proliferation and migration via PD-L1. To investigate whether the effects of Hsp90 solely depend on the PD-L1 expression, Caski and SiHa cells were transiently transfected with pX458-PD-L1 or the pX458 empty vector using Lipofectamine 3000. As shown in Fig. 5D, PD-L1-deficient Caski and SiHa cells were successfully produced. We found that deficiency of PD-L1 did not affect the level of Hsp90, HER2, p-AKT and AKT (Fig. 5E). Overexpression of Hsp90 in PD-L1-deficient Caski and SiHa cells could up-regulate the levels of HER2 and p-Akt, but not affect the level of PD-L1 (Fig. 5E). Furthermore, cell proliferation and migration of PD-L1-deficient Caski and SiHa cells were significantly reduced compared to wild-type cervical cancer cells (Fig. 5F, G). The proliferation and migration of PD-L1-deficient Caski and SiHa cells overexpressed Hsp90 were slightly up-regulated (Fig. 5F, G), indicating that Hsp90 partially dependents PD-L1 pathway to regulate the proliferation and migration of cervical cancer cells.

### *Knockdown of Hsp90 inhibited HPV16*^+^*cervical cancer progression*

**Fig. 6:**
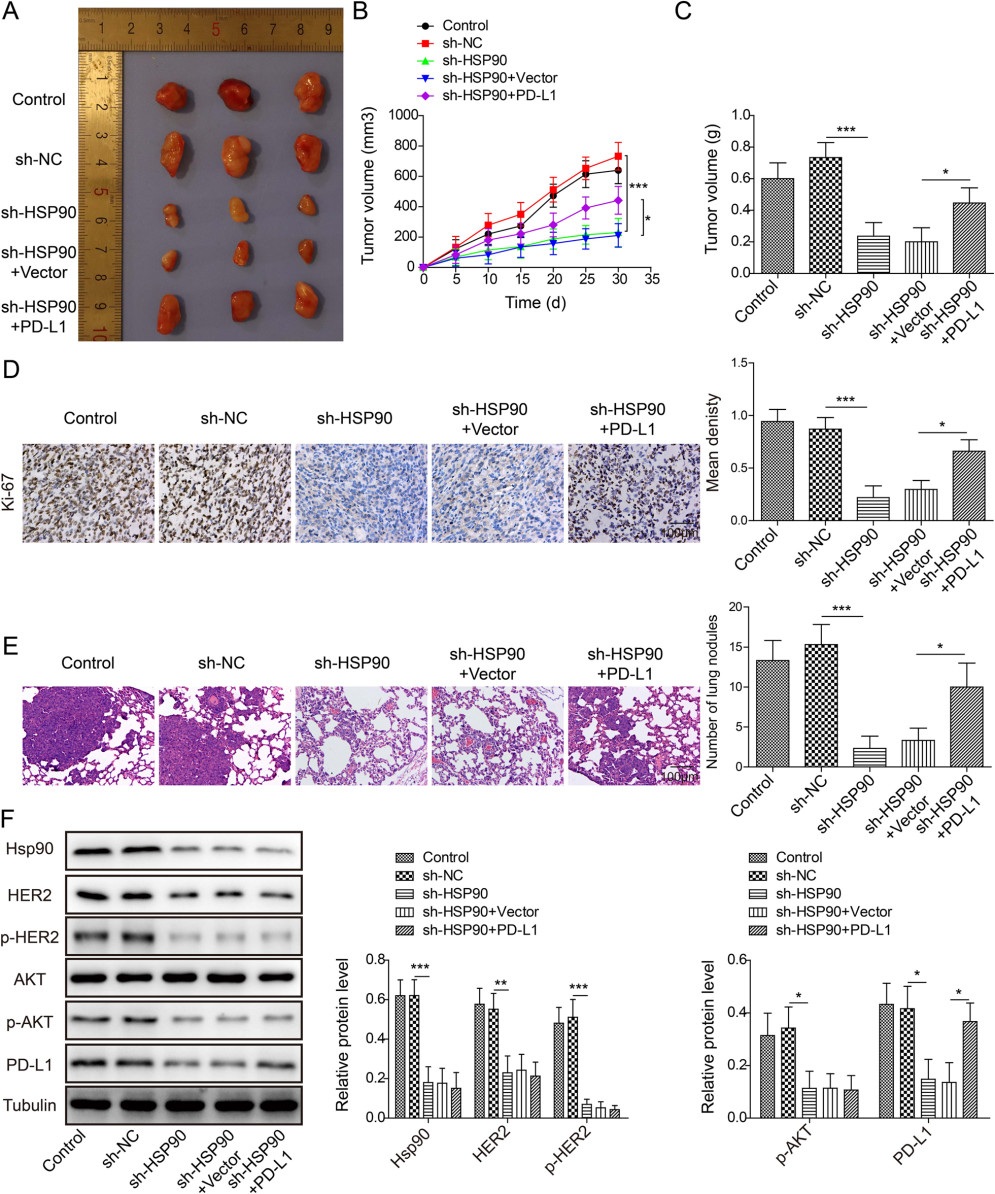
Knockdown Hsp90 inhibited xenograft tumor growth using SiHa cells. **A** gross photos of excised tumors in control, sh-NC, sh-Hsp90, sh-Hsp90+vector and sh-Hsp90+PD-L1 groups. **B** tumor weight and **C** tumor volume. **D** IHC staining by Ki-67 to analyze cell proliferation. **E** H&E staining was used to test lung metastatic nodules. **F** Western blotting image of Hsp90, HER2, p-HER2, AKT, p-AKT. PD-L1 and tubulin expressions. N = 6 in each group, *p < 0.05, **p < 0.01 or ***p < 0.001

We established a mouse xenograft tumor model using transfected SiHa cells. Gross photo of excised tumors demonstrated sh-Hsp90 and sh-Hsp90+vector inhibited tumor formation, whereas sh-Hsp90+PD-L1 had similar tumor size with wild type and sh-NC SiHa (Fig. 6A). The quantification of tumor weight and volume showed significant lower tumor weigh and volume in sh-Hsp90 group and sh-Hsp+vector, and sh-Hsp90+PD-L1 almost restored the tumor growth to the similar level as the wild type and sh-NC control (Fig. 6B, C). H&E staining showed that cell proliferation staining Ki-67 and lung metastatic nodules were reduced in the sh-Hsp90 group compared with the control and sh-NC groups (Fig. 6D, E). However, tumor cell proliferation and lung metastatic nodules increased in sh-Hsp90+PD-L1 group (Fig. 6D, E). Western blotting results showed sh-Hsp90 and sh-Hsp90+vector significantly inhibited Hsp90, HER2, p-HER2, p-AKT and PD-L1 expressions as compared to the control and sh-NC, while sh-Hsp+PD-L1 only restored PD-L1 expression (Fig. 6F), indicating PD-L1 was the downstream of Hsp90/HER2/PI3K/AKT in HPV16^+^ cervical cancer. Results above demonstrated knockdown of Hsp90 had promising tumor-suppressive effect on HPV16^+^ cervical cancer progression.

## Discussion

Cervical cancer is the 4th most common cancer that effects over 570,000 women in 2018 according to World Health Organization (Arbyn et al. 2020). HPV16 is the predominant cancer-causing strain that is responsible for over 50% cervical cancers. In this study, we characterized the expression of Hsp90 in HPV16^+^ cervical cancer tissues, cell lines and xenograft tumor models, and investigated the therapeutic effect of Hsp90 knockdown and its molecular mechanism. We found Hsp90 knockdown significantly inhibited Caski and SiHa cell proliferation and migration in vitro, and suppressed tumor growth in vivo. The underlying mechanism we discovered in this study is Hsp90 knockdown suppressed HER2/PI3K/AKT pathway and PD-L1 expression, leading to inhibit cancer cell proliferation and migration.

Cancer cells express high level of Hsp90, which chaperones and stabilizes oncogenic proteins (Calderon et al. 2019; Liu et al. 2020). Inhibition of Hsp90 has been investigated preclinically in various cancer types including non-small cell lung cancer (Codony-Servat et al. 2019), colorectal cancer (Kryeziu et al. 2019), pancreatic cancer (Daunys et al. 2019), etc. In cervical cancer, Hao et al. conducted a clinical study and found Hsp90 expressed in over 77% of the cervical cancer tissues. They also reported Hsp90 expression in cervical cancer tissues was positively correlated with HPV16 infection (Hao et al. 2010). Several approaches have been made to alter Hsp90 expression in cervical cancer cells. Xu et al. reported miRNA-361 directly targeted Hsp90 to suppress its expression, leading to higher survival rate in cervical cancer patients (Xu et al. 2020). Hu et al. used Hsp90 inhibitor SNX2112 to treat human cervical cancer cells, and caused TRAIL-induced apoptosis of human cervical cancer cells (Hu et al. 2019). In our study, we investigated the Hsp90 expression in clinical samples, and confirmed high Hsp90 expression in HPV16^+^ cervical cancer tissues. Knockdown of Hsp90 inhibited proliferation and migration of Caski and SiHa cells, suggesting our findings are consistent with previous studies in this field (Fu et al. 2020).

PD-L1 plays a major role in suppressing adaptive immune system activation in tumor environment (Juneja et al. 2017; Wang et al. 2017). Binding of PD-L1 to PD-1 can inhibit anti-tumor immunity by counteracting T cell-activating signals (Juneja et al. 2017; Wang et al. 2017). Thus, expression of PD-L1 on tumor cells and other cells in the tumor microenvironment is of major clinical relevance. It was reported that PD-1 could promote lymph node metastasis in cervical cancer via activating integrin β4/SNAI1/SIRT3 axis (Wang et al. 2018). Pei et al. found that α-Cyperone suppressed cervical cancer via the ROS-mediated PI3K/Akt/mTOR signaling pathway and closely related to PD-L1 down-regulation (Pei et al. 2020). Previous study reported *N*-alkyl-hydroxybenzoyl anilide hydroxamates inhibited both Hsp90 and PD-L1 expression lung cancer cells (Mehndiratta et al. 2020), but the regulatory relationship between Hsp90 and PD-L1 is rarely reported. Herein, we found PD-L1 expression was elevated in HPV16^+^ cervical cancer tissues, and positively correlated with Hsp90 expression. PD-L1 expression is induced by activated PI3K/AKT pathway (Gao et al. 2018). Hsp90 stabilizes the PI3K/AKT pathway promoter HER2 expression, activated PI3K/AKT pathway and induce PD-L1 expression. Our results demonstrated knockdown of Hsp90 suppressed HER2 expression, p-AKT activation and PD-L1 expression, leading to the inhibited cervical cancer cell proliferation and migration in vitro and tumor progression in vivo. With the results we obtained, we proposed a modulatory axis of Hsp90/HER2/PI3K/AKT/PDL-1 in cervical cancer cell signaling.

## Conclusions

In conclusion, our study demonstrated Hsp90 is closely associated with HPV16^+^ cervical cancers, and the inhibition of Hsp90 effectively suppressed HPV16^+^ cervical cancer cell proliferation and migration. With mouse xenograft tumor model, we confirmed the tumor-suppressing effect of Hsp90 inhibition. We proved Hsp90 directly modulates HER2 to mediate PI3K/AKT pathway and PD-L1 expression, revealing the underlying molecular mechanism of Hsp90 in cervical cancers. Our data suggested Hsp90 inhibition holds a significant potential in treating HPV16^+^ cervical cancers.

## Data Availability

All data generated or analyzed during this study are included in this article. The datasets used and/or analyzed during the current study are available from the corresponding author on reasonable request.

## Abbreviations

HPV: Human papillomavirus
Hsp90: Heat shock protein 90
HER2: Human epithermal growth factor receptor 2
PD-L1: Programmed death-ligand 1
DMEM: Dulbecco’s Modified Eagle Medium
SDS-PAGE: Sodium dodecyl sulphate–polyacrylamide gel electrophoresis
NC: Negative control
FBS: Fetal bovine serum
ANOVA: Analysis of variance
